# Alteration of immunoproteome profile of *Echinococcus granulosus* hydatid fluid with progression of cystic echinococcosis

**DOI:** 10.1186/s13071-014-0610-7

**Published:** 2015-01-08

**Authors:** Chun-Seob Ahn, Xiumin Han, Young-An Bae, Xiao Ma, Jin-Taek Kim, Huixia Cai, Hyun-Jong Yang, Insug Kang, Hu Wang, Yoon Kong

**Affiliations:** Department of Molecular Parasitology, Sungkyunkwan University School of Medicine and Center for Molecular Medicine, Samsung Biomedical Research Institute, Suwon, 440-746 Korea; Qinghai Province Institute for Endemic Diseases Prevention and Control, Xining, Qinghai China; Department of Microbiology, Graduate School of Medicine, Gachon University, Incheon, Korea; Department of Parasitology, Ewha Womans University, School of Medicine, Seoul, Korea; Department of Molecular Biology and Biochemistry, School of Medicine, Kyung Hee University, Seoul, Korea

**Keywords:** *Echinococcus granulosus*, Cystic echinococcosis, Hydatid fluid, Immunoproteome, Antigen B, Antigen 5, Stage-specificity

## Abstract

**Background:**

Cystic echinococcosis (CE), caused by *Echinococcus granulosus* metacestode, invokes a serious public health concern. Early diagnosis has great impacts on reduction of disability-adjusted life years. Several antigen B-related molecules (EgAgB; EgAgB1-5) are known to be immunopotent, but detection of EgAgB is variable in many patients and may not allow reliable interpretation of its immunological relevance. More importantly, the immunoproteome profile of hydatid fluid (HF) has not been addressed.

**Methods:**

We conducted a proteome analysis of the HF of a single fertile cyst of CE1 and CE2 stages through two-dimensional electrophoresis (2-DE). Each protein spot was analyzed by matrix-assisted laser desorption/ionization time-of-flight mass spectrometry **(**MALDI-TOF-MS). We subsequently determined the immunoproteome profile employing patient sera of entire disease spectrum from CE1 to CE5 stages.

**Results:**

We identified 40 parasite proteins, of which EgAgB (28 spots) and antigen 5 (EgAg5; 5 molecules) were abundant. EgAgB proteoforms constituted the majority, mostly EgAgB1 (24 spots), followed by EgAgB2 and EgAgB4 (2 spots each). EgAgB3 was detected only by liquid chromatography-MS/MS. EgAgB5 was not recognized. We also detected 38 host proteins, which were largely composed of serum components, antioxidant/xenobiotic enzymes, and enzymes involved in carbohydrate metabolism. CE1 and CE2 HF exhibited comparable spotting patterns, but CE2 HF harbored greater amounts of EgAgB and EgAg5 complexes. CE sera demonstrated complicated immune recognition patterns according to the disease progression; CE2 and CE3 stages exhibited strong antibody responses against diverse EgAgB and EgAg5 proteoforms, while CE1, CE4, and CE5 stages mainly reacted to EgAg5 and cathepsin B. Patient sera of alveolar echinococcosis (AE) cross-reacted with diverse EgAgB isoforms (36%). EgAg5 and cathepsin B also demonstrated cross-reactions with sera from neurocysticercosis and sparganosis.

**Conclusions:**

Our results demonstrated that detection of a single defined molecule may not properly diagnose CE, since specific immunodominant epitopes changed as the disease progresses. Immunoproteome analysis combined with imaging studies may be practical in the differential diagnosis of CE from AE and other cystic lesions, as well as for staging CE, which are pertinent to establish appropriate patient management.

**Electronic supplementary material:**

The online version of this article (doi:10.1186/s13071-014-0610-7) contains supplementary material, which is available to authorized users.

## Background

Cystic echinococcosis (CE), caused by *Echinococcus granulosus* metacestode, is one of the most deleterious helminthic diseases of humans and livestock. CE is detected worldwide, but it is more prevalent in the nomadic areas of Central and Middle Asia, Eastern Europe, Africa, Australia, South America, and northwestern China [1-3]. Approximately 4 million people are infected and another 40 million are at risk of infection annually [4]. Humans are infected by incidental contract with the eggs in association with dog rearing environments. Oncospheres hatched from the eggs are activated in the small intestine and subsequently penetrate the intestinal wall to enter the circulation. They mostly egress in the liver and lung, and grow slowly to hydatid fluid (HF)-filled cyst, in which many protoscoleces and daughter cysts develop [5].

Clinical manifestations of CE are rarely present until large cyst(s) crowd the affected organs/tissues. Approximately 60-88% of patients with cysts less than 7.5 cm in diameter manifest no discernible symptoms [6,7]. Most CE cases are diagnosed ages between the third and fifth decades, but the highest morbidity has been observed in young patients under the first two decades [8]. Therefore, early detection significantly reduces morbidity and mortality associated with CE, which remains with challenging issues. The diagnosis of CE largely depends on imaging scans and serological tests. However, imaging diagnosis modalities, such as ultrasonography (US), computed tomography (CT), and magnetic resonance imaging (MRI), necessitate differential diagnosis from alveolar echinococcosis (AE), cystic lesions, liver cirrhosis, and primary hepatocellular carcinoma [5,9]. Staging of CE is also ambiguous in many cases. In such complex situations, serodiagnosis provides additional evidence for CE in patients with hepatic/pulmonary lesions and is beneficial to screen and diagnose patients in endemic areas [10].

Several component proteins of HF, such as diverse isoforms of antigen 5 (EgAg5) and antigen B (EgAgB) are known to be potent diagnostic antigens, among which native and recombinant EgAgBs are reliable and reproducible antigens that allow for better diagnostic performance [5,11,12]. EgAgB is a thermostable, macromolecular multifunctional lipoprotein encoded by a multigene family [13]. At least 10 genes in five subclasses are differentially expressed from the parasite [14]. Its functions include elastase inhibition, augmentation of TH2-biased immune responses, and sequestration of hydrophobic substances [11,15-17]. However, it has not been critically determined whether specific antibodies against EgAgB are detected in both the active and chronic stages of the disease or whether EgAgB shows species-specificity. Demonstration of specific antibodies against EgAgB varies from patient to patient, which does not allow clinical interpretation of its immunological significance [5,11,16,18,19]. More importantly, aspects of the immunoproteome profile, such as global antibody responses and alteration of specific antigens in relation to disease characteristics have not been investigated.

In the present study, we analyzed the proteome profile of a single fertile cyst of CE1 and CE2 stages through matrix-assisted laser desorption/ionization time-of-flight mass spectrometry (MALDI-TOF-MS), and identified 40 parasite proteins, in which EgAgB (28 spots) and EgAg5 (5 molecules) were abundant. We subsequently determined their antigenic properties along with CE progression, as well as their cross-reactions with sera of AE and other diseases. Our results demonstrate that immune recognition patterns change with disease characteristics, and further suggest that simultaneous monitoring of the immunoproteome profile and imaging scans might be required to properly diagnose and appropriately manage CE patients.

## Methods

### Parasite samples

Hepatic CE cysts were obtained from the naturally infected sheep slaughtered in Xining, Qinghai Province, China. Individual cysts were examined by US, and fertile CE1 and CE2 cysts were independently collected. After careful decortication, the cyst surface was washed with physiological saline more than 10 times at 4°C. Each HF was aseptically drawn from intact cysts in the presence of a protease inhibitor cocktail (one tablet/25 ml, Roche, Germany) and centrifuged at 20000 × g for 1 h at 4°C. Supernatants were concentrated by lyophilization and stored at −80°C until use.

### Study subject and serum samples

Sera of 59 hepatic CE patients (37.1% male), whose disease statues were defined by US [3], were studied. Patients of CE1 stage (*n* = 11) showed unilocular hypodense cyst(s) with a double line sign. CE2 patients (*n* = 12) presented with multivesicular, multiseptated cysts with detached endocysts. Patients of CE3 (*n* = 12) demonstrated unilocular cysts with daughter cysts that contained a solid cyst matrix or detached endocyst. CE4 patients (*n* = 13) showed typical canalicular structure (ball of wool appearance). CE5 patients (*n* = 11) presented with heavily degenerative cyst(s) with calcified wall. Involvement of the right lobe was frequent (71%). Eleven patients (18.6%) had multiple cysts. Most patients (85%) did not complain of specific symptoms/signs. Their demo-epidemiological profile, cyst locations, and representative US findings are summarized in Additional file 1. In addition, this study included 10 sera per each of the following groups: neurocysticercosis, AE, sparganosis, pathology-proven primary hepatocellular carcinoma, and healthy controls, all of whom denied any possible exposure to protozoan and helminthic infections [5,20]. Study protocol was approved by the Institutional Review Committee of Qinghai Province Institute for Endemic Diseases Prevention and Control (protocol no. 2013-7-22). The study was presented to the local health authorities, community leaders, and patients to ensure their acceptance and collaboration. Written informed consent was obtained from all patients. In the case of illiterate patients, consent was obtained by verbal notification.

### Two-dimensional electrophoresis (2-DE) and immunoblotting

Each HF (200 μg) mixed with rehydration buffer (6 M urea, 2 M thiourea, 2% CHAPS, 0.4% dithiothreitol [DTT], 0.5% immobilized pH gradient [IPG] buffer and 0.002% bromophenol blue [BPB]), was cup-loaded on IPG strips (pH 6–11, 13 cm; GE Healthcare, Piscataway, NJ, USA), focused for a total of 25 kVh, processed with 15% SDS-PAGE (160 × 160 × 1.5 mm), and stained with Coomassie Brilliant Blue G-250 (CBB). For immunoblotting, proteins separated by 2-DE were electroblotted to nitrocellulose membranes (Santa Cruz, Dallas, TX, USA) for 8 h at 4°C. The membranes were blocked with Tris-buffered saline (100 mM, pH 8.0) containing 3% skim milk and 0.1% Tween-20 for 1 h. The blots were incubated with individual and/or pooled serum overnight (1:1000-dilution) at 4°C and sequentially with horse-radish peroxidase (HRP)-conjugated anti-human IgG (Cappel, Solon, OH, USA) for 2 h (1:4000-dilution). For quantitative analysis, all signals were detected with SuperSignal Chemiluminescence (ECL; GE Healthcare) after a 2 min exposure. Images were digitalized with an Umax Image Scanner (Dallas, TX, USA) and analyzed employing Progenesis software (Nonlinear Dynamics, Newcastle, UK). The individual spots, whether stained or immunoblotted, were quantified by calculation of the spot volume using the total spot volume normalization method, multiplied by the total area of all the spots.

### Matrix-assisted laser desorption/ionization time-of-flight mass spectrometry (MALDI-TOF-MS) and nano-liquid chromatography-electrospray ionization/multi-stage MS (nano-LC-ESI-MS/MS)

Two different sets of in-gel trypsin-digested protein spots from each CE1 and CE2 HF were independently applied using the AB SCIEX TOF/TOF 4800 Plus System (AB SCIEX, Framingham, MA, USA). Internal standards were tryptic autodigestion peaks (*m/z* = 842.5099 and 2211.1046). Monoisotopic peptide masses were selected between 600 and 3500 Da. The proteins were identified by peptide mass fingerprint (PMF) with a Mascot server. Mass tolerance was ± 50 ppm. One missed cleavage site was allowed. Identification was accepted when PMF harbored at least two identified peptides (>99% probability).

For nano-LC-ESI-MS/MS, CE1 and CE2 HF were separated by 15% reducing SDS-PAGE and visualized with CBB staining. Each band corresponding to 24 kDa was cut and sliced into fragments from independent gels. Disulfide bonds were reduced with 10 mM DTT. After alkylation, gels were dehydrated, trypsin-digested, and dried in a vacuum evaporator (MIVAC DUO, Genevac, Ipswich, UK). Analysis was conducted employing a model 1200 nano-flow system (Agilent Technologies, Palo Alto, CA, USA) connected to a LTQ linear ion trap mass spectrometer (Thermo Electron, San Jose, CA, USA). The reversed phase capillary column was 12 cm in length, 75 mm in inner diameter, and in-house packed with 5 μm 200 Å pore-sized Magic C18AQ beads (Michrome BioResources, Auburn, CA, USA). The peptides were eluted in a linear gradient from 10 to 40% acetonitrile over 65 min. MS survey was scanned for 300–2000 *m/z* with three data-dependent MS/MS scans of isolation width (1.5 *m/z*), normalized collision energy (25%), and dynamic exclusion duration (180 sec). MS data was generated in RAW file format (Thermo Scientific) using the Xcalibur 1.4 with Tune 1.0. Peptide peaks were introduced into MS/MS ions search within a Mascot server (http://www.matrixscience.com). Mass values were selected using monoisotopic masses. Peptide and MS/MS tolerances were ± 1.2 and ± 0.6 Da. Protein identifications of individual ions scores > 43 were considered as significant or to demonstrate extensive homology (*p* < 0.05).

Cysteine carbamidomethylation and methionine oxidation were considered during the analyses. MS database search was performed on the NCBI database (http://www.ncbi.nlm.nih.gov). Signal peptide sequences were predicted by SignalP 4.1 (http://www.cbs.dtu.dk/services/SignalP/) and PSORT (http://psort.hgc.jp/form.html).

## Results

### Proteomic analysis of *E. granulosus* HF collected from different CE stages reveals similar spotting patterns, but CE2 HF harbored enriched EgAgB and EgAg5 proteoforms

**Figure 1:**
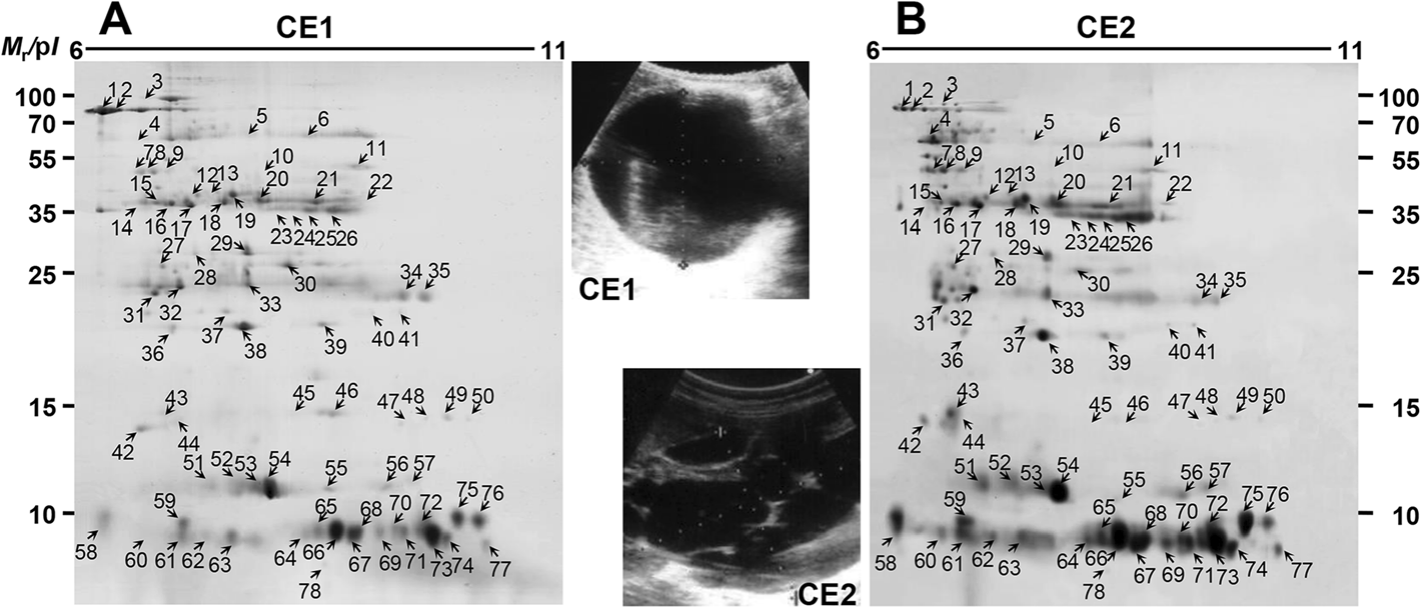
**Electrophoretic profile of** ***E. granulosus*** **HF collected from sheep livers.** HF (200 μg proteins) harvested from single fertile cyst of CE1 **(A)** and CE2 **(B)** stages were electrofocused on IPG strips (pH 6–11) and resolved by 15% SDS-PAGE. The gels were stained with CBB. The protein spots were subjected to in-gel trypsin digestion and processed with MALDI-TOF-MS. Proteins identified are marked by Arabic numbers (1–78) and are summarized in Table 1 and Additional file 2. Representative ultrasonographic images of CE1 and CE2 cysts are shown. *M*_r_, molecular weights in kDa; p*I*, isoelectric point.

In order to comparatively delineate HF proteins of different CE stages, we determined the protein profile of a single fertile CE1 and CE2 cyst. As shown in Figure 1A and B, equivalent spotting patterns were evident, but CE2 HF harbored more EgAgB than CE1 HF. The expression pattern of EgAg5 was also shown to be upregulated in CE2 HF (see also Table 1). We identified 78 protein spots (*p* < 0.01); 40 *Echinococcus* and 38 host proteins. All the *Echinococcus* proteins were concomitantly identified; they typically segregated into diverse isoforms of EgAgB and EgAg5. EgAgB proteoforms appeared to be encoded by six different genes (ACZ51458, ACZ51459, and ACP21247 for EgAgB1; AAP83174 and AAW78457 for EgAgB2; and ACZ51452 for EgAgB4). Among the EgAgB complex, EgAgB1 was the major components comprising 24 molecules between 8 and 16 kDa (spots 43–49, 60–74, 77, and 78). Each of two EgAgB4- (spots 58 and 59) and EgAgB2-related proteins (spots 75 and 76) was recognized. EgAgB3- and EgAgB5-related molecules could not be identified. This result matched well with previous studies in which *EgAgB1*, *EgAgB2*, and *EgAgB4* were abundantly transcribed and expressed among several EgAgB isoforms [14,21]. Five EgAg5 isoforms/variants were detected (spots 21 and 23–26; ADG65665). Other molecules (7 spots) included enzymes involved in carbohydrate metabolism (5 molecules), such as phosphoenolpyruvate carboxykinase (spot 2), citrate synthase (spot 10), phosphoglycerate kinase (spot 11), fructose 1,6 biphosphate aldolase (spot 22), and triophosphate isomerase (spot 32), and proteolytic cathepsin B (2 molecules; spots 7 and 9) (Table 1 and Additional file 2).

**Table 1:** **Identification of** ***E. granulosus*** **proteins in CE1 and CE2 cysts by MALDI-TOF-MS**

**Spot no(s).**	**Theoretical** ***M***_**r**_ **/p** ***I***	**Accession no**	**Description** ^**a**^	**SP** ^**b**^
2	55590/8.39	CBH36490	Phosphoenolpyruvate carboxykinase	-
7, 9	39841/6.82	EgrG_000790200	Cathepsin B	+
10	51345/8.26	EgrG_001028500	Citrate synthase	-
11	44227/8.03	EUB59398	Phosphoglycerate kinase	-
21, 23-26	33316/9.02	ADG65665	38 kDa antigen 5	-
22	39666/8.31	EgrG_000905600	Fructose 1,6 bisphosphate aldolase	-
32	27123/6.60	EgrG_000416400	Triosephosphate isomerase	-
43-49, 60–71, 74, 77, 78,	6232/8.31	ACZ51459	EgAgB1/1	+
72	5348/9.30	ACP21247	EgAgB1 subunit, partial	+
73	7223/9.23	ACZ51458	EgAgB1	+
58, 59	8337/6.78	ACZ51452	EgAgB4/1	+
75	9282/9.38	AAW78457	EgAgB2	+
76	9314/9.38	AAP83174	EgAgB subunit 2	+

^a^Protein names were adopted from the GenBank database. MALDI-TOF-MS analysis was duplicated for each HF sample from CE1 and CE2 stages.

^b^The presence of a signal peptide (SP) sequence was predicted by SignalP 4.1 and PSORT.

We also identified 38 host-derived ovine proteins. These molecules mainly consisted of serum components (serotransferrin, carbonic anhydrase, and immunoglobulin and hemoglobin families). In addition, antioxidant/detoxification/xenobiotic proteins (selenium-binding protein 1, dihydrodiol dehydrogenase 3, peroxiredoxin-1, and glutathione transferases), enzymes involved in carbohydrate (sorbitol dehydrogenase and alcohol dehydrogenase) and amino acid metabolism (fumarylacetoacetase), fatty-acid-binding proteins (lipid transporter), phosphatidylethanolamine-binding protein (signaling molecule), oxidoreductase (glycerol-3-phosphate dehydrogenase and carbonyl reductase), sugar-binding galectin-3, and γ-tubulin were detected (Additional file 2).

### Immune recognition profile of HF against sera of different CE stages are altered along with CE progression and differed from one another

**Figure 2:**
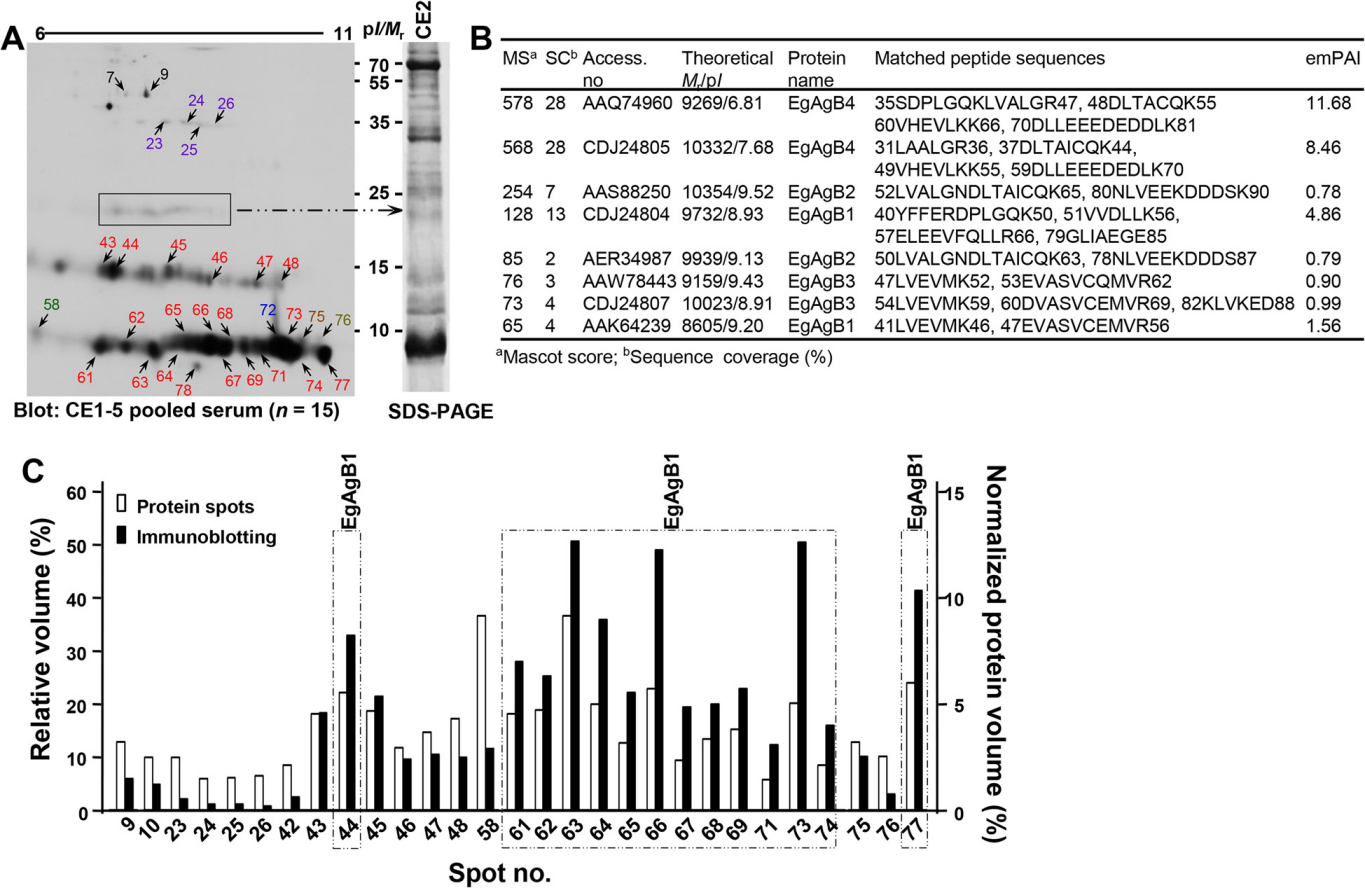
**Global immunoproteome characteristics of CE2 HF. (A)** Immunoproteome analysis of HF against pooled sera of different CE stages. The patients were classified by typical US findings [4]. CE2 HF was separated by 2-DE, transferred to nitrocellulose membranes, and probed with pooled CE1-CE5 sera (*n* = 3 per group) as described in the Methods section. Immunoreactions of EgAg5 (purple), EgAgB1 subunit (blue), EgAgB1 (red), EgAgB subunit 2 (khaki), EgAgB2 (brown), and EgAgB4 (green) are indicated. All signals were detected by ECL after 2 min exposure. Spots at ca. 24 kDa could not be identified on 2-DE gel, but showed immunoreactivity (box, left panel). Each corresponding band from CE1 and CE2 HF was independently analyzed by nano-LC-ESI-MS/MS. **(B)** Identification of proteins within box in A by PMF. **(C)** Immunoreactivity of each spot in Figure 1A (left panel) was compared to the volumes of the corresponding protein spots. The open bars indicate the relative volumes (left Y-axis) and normalized protein volumes (right Y-axis) in the CBB stained gels. The solid bars indicate the relative volumes of the immunoblot.

The EgAgB complex has been well known to induce specific antibody responses against CE sera [1,5,10,11,16], while analysis of the global immunoproteome profile and changes of specific epitopes along with CE characteristics have not been explored [19]. In order to establish a global immunoproteome profile, we observed the immunorecognition pattern of HFs of sheep CE1 and CE2 cysts by using pooled patient serum from CE1-CE5 stages (*n* = 3 per group). These HFs demonstrated an immunoreactivity profile similar to each other, but CE2 HF exhibited more distinct patterns than CE1 (left panel, Figure 2A; other data not shown). We used CE2 HF to compile the CE immunoproteome.

When blots containing CE2 HF were probed with a pooled CE patient serum, cathepsin B (spots 7 and 9), EgAg5 (spots 23–26), and diverse EgAgB proteoforms demonstrated strong antibody reactions in a highly complicated fashion. Interestingly, five to seven spots located at approximately 24 kDa, which could not be identified through MALDI-TOF-MS analysis, displayed positive reactions (box, Figure 2A). We analyzed the corresponding band (right panel, Figure 2A) by LC-ESI-MS/MS and recognized that this band contained EgAgB1, EgAgB2, EgAgB3, and EgAg4 isoforms (Figure 2B).

Among the diverse EgAgB proteoforms, EgAgB1 (spots 44, 61–69, 71, 73, 74, and 77) revealed high antibody capturing activity, which comprised 55.1% of the total volume of the proteins and 80.2% of the total volume of the antigenic spots (dotted-boxes, Figure 2C). Other EgAgB subunits, such as EgAgB2, EgAgB3, and EgAgB4 exhibited minimal antibody reactivity. This result convincingly demonstrated that EgAgB1 isoforms constituted immunodominant antigens.

**Figure 3:**
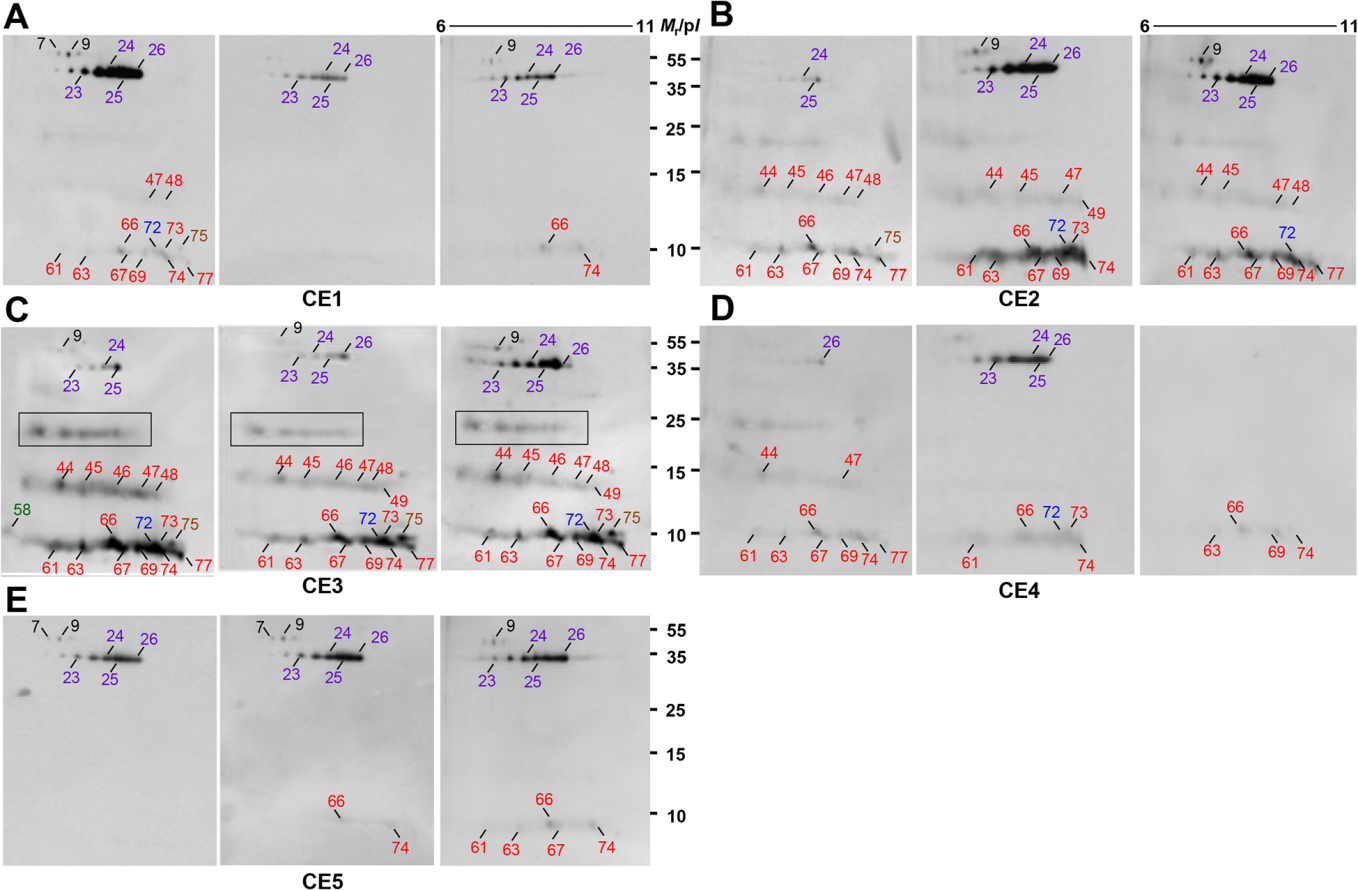
**Changes of immunoproteome profile according to CE progression.** Blots containing CE2 HF were probed with individual CE1-CE5 sera as described in the Methods section. Markings are the same as in Figure 2A. Panels **A-E** show representative immunoblot images from respective disease stages (CE1-CE5). *M*_r_, molecular weights in kDa; p*I*, isoelectric point.

We subsequently determined that the alteration of immunoreactive spots in accordance with CE involution (Figure 3). When we probed individual CE1 sera, immunoreactions against EgAg5 (spots 23–26) and cathepsin B (spot 9) were fairly high (36.4-72.7%). Conversely, EgAgB1 isoforms, such as spots 47, 48, 61, 63, 66, 67, 69, 72–74, and 77 showed relatively weak responses (9.1-54.5%) (panel A). CE2 patient sera demonstrated dissimilar immune recognition patterns as compared to those of CE1; reactions against EgAgB subunits, especially against 8 kDa isoforms became evident with growing frequency (75-100%), with the exception of spot 61 (33.3%). Cathepsin B (spot 9) and EgAg5 (spots 23–26) also showed positive responses (75–91.7%) (panel B). As the disease progressed to the transitional CE3 stage, a variety of EgAgB isoforms (spots 44–48, 61, 63, 66, 67, 69, 72–75, and 77) exhibited prominent antibody responses (75-100%). EgAg5 (spots 23–26) also revealed positive reactions. In this stage, five to seven spots located at approximately 24 kDa, which were identified as various EgAgB isoforms (Figure 2B), demonstrated positive reactions as well (panel C). As the disease progressed to inactive CE4 and CE5 stages, the immunoproteome profile regressed toward that observed in CE1. Cathepsin B (spot 9) and 38 kDa EgAg5 subunits (spots 23–26) retained their responsiveness (30.8-85.7%), while EgAgB proteoforms revealed significantly decreased antibody capturing activity (0–63.6%) (panels D and E).

### Assessment of cross-reactivity

**Figure 4:**
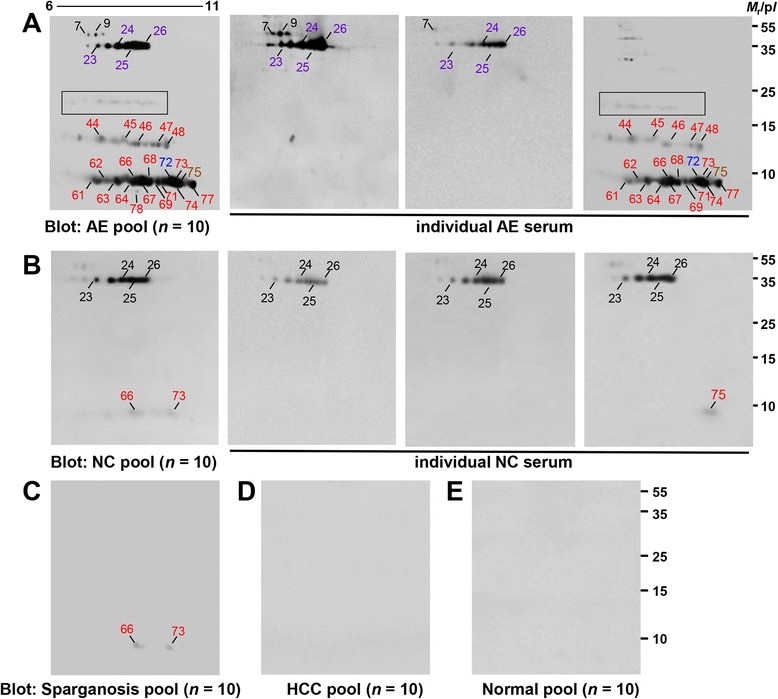
**Cross-reactivity with other pathological conditions and normal controls.** Blots containing CE2 HF were reacted with individual/pooled (*n* = 10 per group) serum of AE **(A)**, neurocysticercosis **(B)**, sparganosis **(C)**, primary hepatocellular carcinoma **(D)**, and normal controls **(E)**. EgAgB1 shows cross-reactivity with AE patient sera (boxes in panel **A**). Markings are the same as in Figure 2A. AE, alveolar echinococcosis; NC, neurocysticercosis; HCC, primary hepatocellular carcinoma. *M*_r_, molecular weights in kDa; p*I*, isoelectric point.

We determined whether or not the proteins that showed high immunoreactivity against CE sera cross-reacted with heterologous infection sera and hepatocellular carcinoma patient sera. When pooled serum of AE patients (*n* =10) was probed with CE2 HF, cathepsin B (spots 7 and 9), EgAg5 (spots 23–26), EgAgB2 (spot 75), and some EgAgB1 isoforms (spots 44–48, 61–64, 66–69, 71–74, and 77) exhibited serological cross-reactions. Further analyses employing individual AE sera demonstrated cross-reactivity of EgAg5 (70-80%) and EgAgB1 (20-50%) with a complicated pattern (Figure 4A). Pooled serum (each of *n* = 10) or individual sera of neurocysticercosis and sparganosis also exhibited weak cross-reactions with certain isoforms of EgAg5 (spots 23–26) and EgAgB (spots 66, 73, and 75) (Figure 4B and C). Serological cross-reactions between CE and AE may be inherent to some extent due to the highly conserved sequence identity between EgAgBs and EmAgBs (78.8-94.2%), except for the antigen B3 series (36.5%) (data not shown), which did not invoke a significant antibody response against CE patients. Primary hepatocellular carcinoma patient sera and sera of normal controls did not show any detectable cross-reactivity (Figure 4D and E). Table 2 summarizes the evaluation of sensitivity and specificity of important diagnostic spots by 2-DE/immunoblotting.

**Table 2:** **Responsiveness of major antigenic spots of** ***E. granulosus*** **HF**

**Spot no. (Description** ^**a**^ **)**	**No. of positive (% sensitivity)**						**No. of negative (% specificity)**			
	**CE1 (** ***n*** **= 11)**	**CE2 (** ***n*** **= 12)**	**CE3 (** ***n*** **= 12)**	**CE4 (** ***n*** **= 13)**	**CE5 (** ***n*** **= 11)**	**Overall (** ***n*** **= 59)**	**AE (** ***n*** **= 10)**	**NC (** ***n*** **= 10)**	**Others** ^**b**^ **(** ***n*** **= 30)**	**Overall (** ***n*** **= 50)**
9 (Cathepsin B)	5 (45.5)	9 (75 )	12 (100)	4 (30.8)	6 (85.7)	**36 (61)**	3 (30)	9 (90)	30 (100)	**42 (84)**
23 (38 kDa EgAg5)	4 (36.4)	11 (91.7)	12 (100)	7 (53.9)	9 (81.8)	**43 (72.9)**	2 (20)	6 (60)	30 (100)	**38 (76)**
24 (38 kDa EgAg5)	5 (45.5)	10 (83.3)	12 (100)	8 (61.5)	8 (72.7)	**43 (72.9)**	2 (20)	6 (60)	30 (100)	**38 (76)**
25 (38 kDa EgAg5)	6 (54.5)	10 (83.3)	12 (100)	8 (61.5)	8 (72.7)	**44 (74.6)**	2 (20)	6 (60)	30 (100)	**38 (76)**
26 (38 kDa EgAg5)	8 (72.7)	10 (83.3)	12 (100)	7 (53.8)	9 (81.8)	**46 (80.0)**	3 (30)	6 (60)	30 (100)	**39 (78)**
44 (EgAgB1/1)	0 (0)	10 (83.3)	10 (83.3)	7 (53.8)	0 (0)	**27 (45.8)**	8 (80)	10 (100)	30 (100)	**48 (96)**
45 (EgAgB1/1)	0 (0)	9 (75)	10 (83.3)	6 (46.2)	0 (0)	**25 (42.4)**	7 (70)	10 (100)	30 (100)	**47 (94)**
46 (EgAgB1/1)	0 (0)	10 (83.3)	11 (91.7)	5 (38.5)	0 (0)	**26 (44.1)**	8 (80)	10 (100)	30 (100)	**48 (96)**
47 (EgAgB1/1)	4 (36.4)	10 (83.3)	10 (83.3)	6 (46.2)	0 (0)	**30 (50.8)**	8 (80)	10 (100)	30 (100)	**48 (96)**
48 (EgAgB1/1)	3 (27.3)	9 (75)	10 (83.3)	5 (38.5)	0 (0)	**27 (45.8)**	7 (70)	10 (100)	30 (100)	**47 (94)**
61 (EgAgB1/1)	1 (9.1)	4 (33.3)	9 (75)	5 (38.5)	3 (27.3)	**22 (37.3)**	6 (60)	10 (100)	30 (100)	**46 (92)**
63 (EgAgB1/1)	4 (36.4)	9 (75)	12 (100)	7 (53.9)	6 (54.5)	**38 (64.4)**	7 (70)	10 (100)	30 (100)	**47 (94)**
66 (EgAgB1/1)	6 (54.5)	10 (83.3)	12 (100)	11 (84.6)	5 (45.5)	**44 (74.6)**	5 (50)	8 (80)	28 (93.3)	**41 (82)**
67 (EgAgB1/1)	5 (45.5)	9 (75)	12 (100)	5 (38.5)	2 (18.2)	**33 (55.9)**	7 (70)	10 (100)	30 (100)	**47 (94)**
69 (EgAgB1/1)	5 (45.5)	9 (75)	12 (100)	7 (53.8)	0 (0)	**33 (55.9)**	6 (60)	10 (100)	30 (100)	**46 (92)**
72 (EgAgB1 subunit)	6 (54.5)	10 (83.3)	12 (100)	10 (76.9)	7 (63.6)	**45 (76.3)**	7 (70)	10 (100)	30 (100)	**47 (94)**
73 (EgAgB1/1)	5 (45.6)	11 (91.7)	12 (100)	4 (30.8)	0 (0)	**32 (54.2)**	5 (50)	8 (80)	29 (96.7)	**42 (84)**
74 (EgAgB1/1)	4 (36.4)	10 (83.3)	12 (100)	5 (38.5)	4 (36.4)	**32 (54.2)**	7 (70)	10 (100)	30 (100)	**47 (94)**
75 (EgAgB2)	4 (36.4)	10 (83.3)	12 (100)	5 (38.5)	4 (36.4)	**35 (59.3)**	5 (50)	7 (70)	30 (100)	**42 (84)**
77 (EgAgB1/1)	4 (36.4)	12 (100)	12 (100)	5 (38.5)	6 (54.5)	**39 (66.1)**	6 (60)	10 (100)	30 (100)	**46 (92)**

^a^Protein names were adapted from the GenBank database.

^b^Others included sparganosis, primary hepatocellular carcinoma, and normal controls (each of *n* = 10).

## Discussion

In this study, we have undertaken a comprehensive proteome analysis of CE1 and CE2 HF and identified 78 individual protein spots (40 *Echinococcus* and 38 host proteins), in which EgAgB (28 species) and EgAg5 (5 molecules) were most abundantly recognized. Among the EgAgB series, EgAgB1 represented the most profoundly expressed proteoforms regardless of CE status, which was consistent with previous proteomic analysis of ovine CE HF [21]. We subsequently determined the antigenic properties of the respective proteoforms along with CE progression and established a global CE immunoproteome profile for the first time.

Our results demonstrated that immune recognition patterns of CE patients were altered along with the disease progression and they differed from one another. In both the early CE1, and the chronic inactive CE4 and CE5 stages, EgAg5 played a major role in inducing antibody responses. This result suggests that posttranslationally modified, externally exposed phosphorylcholine moieties of the EgAg5 (a major antigenic epitope), might be readily accessible to the immune system [12,22], although its specificity is fairly low. EgAg5 is a high-molecular-weight glycoprotein complex (>500 kDa) consisting of 57 and 67 kDa under reducing conditions, and is dissociated into 22 and 38 kDa subunits [22]. The protein has been widely used for CE immunodiagnosis, while other studies have demonstrated that EgAg5 cross-reacted with the patient sera of other helminthic infections [12,23]. Indeed, several EgAg5 molecules showed cross-reactions with AE and neurocysticercosis sera (Figure 4 and Table 2). In the early stage of CE, the immunoreactivity of EgAgB isoforms was reasonably weak. Previous studies involving animal CE immunodiagnosis revealed that EgAgB demonstrated quite low sensitivity [24,25]. This result strongly suggested that these animals, even infected, might be in an early stage of CE since the cattle slaughtered in abattoirs are mostly young. Sera from these animals might not respond to EgAgB as shown in this study. These collective results indicated that EgAgB proteoforms may not be applicable for serodiagnosis of early and chronic stages of CE.

As cyst(s) develops and progresses into the active stage, a substantially expanded germinal layer synthesizes and secretes copious quantities of several tens of EgAgB, and induces antibody responses. In the transitional CE3 period, previously secreted diverse EgAgB molecules continuously stimulate the immune system. A partially degenerative germinal layer might maintain its capacity to produce and secret EgAgBs, which results in the strong antibody responses, as is seen in this study. Among several EgAgB proteoforms, EgAgB1 constituted the majority with the strongest antigenicity. When metacestode(s) regressed to chronic inactive stages, EgAgB-enriched HF might be rapidly drained and become absorbed through degenerative membranes. Subsequently, the synthesis of EgAgB might also be attenuated and the antibody responses culminating to EgAgB would become negligible. A similar phenomenon has been observed in neurocysticercosis, which is caused by a phylogentically-close neighbor, the *Taenia solium* metacestode. Expression of a 150-kDa hydrophobic-ligand-binding-protein (HLBP) complex, which invokes specific and strong antibody reactions during the active stage, is drastically down-regulated as the parasite undergoes degeneration by calcification [26,27]. This common pattern of expressional regulation of HLBPs may have significant pathophysiological implications in the long-term survival of larval taeniid, such as *E. granulosus* and *T. solium* metacestodes within host environments, since HLBPs are critical in recruiting essential host lipids, regulating local immune responses, and modulating protein turnover [17,26,27]. This result also raised an intriguing suggestion that blocking the synthesis or inactivating these molecules might be an attractive target to control CE and neurocysticercosis. Molecular mechanisms underlining expressional regulation of several EgAgB proteoforms should form the basis of future studies.

The EgAgB2 appeared to be a reliable antigenic protein for CE serodiagnosis, when the HFs of bovine and human origin were employed as antigens [28]. However, such a potent immunoreactivity of EgAgB2 proteoforms was not observed with ovine CE2 HF in this study. It would be interesting to analyze whether the expressions of different EgAgBs are differentially regulated in different hosts. We could not detect individual EgAgB3-related molecules through 2-DE analysis. These molecules were only identifiable by LC-ESI-MS/MS. Since EgAgB3 is primarily synthesized in the protoscolex [29], our result suggests that, although transcription levels of EgAgB3 are relatively high in the protoscolex [14], secretion of EgAgB3 at protein levels would be minimal, and correlated with the finding that its antigenicity was reasonably weak and was detected only in the active and transitional periods, during which active immune systems are operating (Figure 3). In addition, we were unable to detect EgAgB5 molecules, which harbored similar primary sequences to EgAgB3 molecules. This isoform was indeed shown to be mainly expressed in the immature adult, but not the metacestode, stage [14,21].

CE cyst thrives for over 20 years in immunologically competent hosts. The parasite continuously interacts with the host to defend the cytopathic environment. In addition to diverse EgAgB, HF contains many secretory proteins, which may contribute to parasite survival through the regulation of bioactive molecules [29,30]. Some metabolic enzymes, especially those involved in carbohydrate metabolism, are known to perform several moonlighting activities [31-33]. We identified multifaceted *E. granulosus* protein enzymes, such as phosphoenolpyruvate carboxykinase (maintenance of cytosolic and mitochondrial redox balance, and induction of T-cell immunity), citrate synthase (chaperon activity, growth, and virulence), fructose 1,6 bisphosphate aldolase (cellular adhesion and antioxidant activity), and triosephosphate isomerase (anti-oxidative processes and glutathione redox cycle) [33-36]. In this study, we were able to detect host-derived serum components, antioxidant/detoxification/xenobiotic proteins, enzymes involved in carbohydrate and amino acid metabolism, and fatty-acid-binding proteins, which might be absorbed through transporter-mediated-uptake or endocytosis [26,37]. These proteins might exert their moonlighting effects within the HF. Dynamic molecular biochemical and cellular events might be actively occurring within the HF, and *Echinococcus* might absorb and exploit these molecules to maintain its homeostatic function [36]. Further research is warranted to explicate the biological roles of individual proteins together with the molecular network and cross-talk functions within the HF.

## Conclusions

Early detection of CE may have a great impact on reduction of disability-adjusted life years, because the highest morbidity is observed in young patients under 20 years old [1,5]. Our results convincingly demonstrate that the detection of a single defined molecule may not properly diagnose CE since specific immunopotent antigens are altered according to CE progression. Serological cross-reactions between CE and AE may be inherent to some extent. Surveillance of immunoproteome characteristics combined with imaging scans may be essential to differentiate CE from AE and to clarify CE status. Our data also highlighted the possible biological functions of HF proteins, which might be intimately involved in the homeostatic maintenance and pathophysiological adaptation of the parasite during long-standing infections.

## Additional files

Additional file 1:
**Demographic and clinical features of CE patients enrolled in this study.**
Additional file 2:
**Protein identification of sheep CE1 and CE2 HF following MALDI-TOF-MS analysis.**

## Abbreviations

AE: Alveolar echinococcosis
CBB: Coomassie brilliant blue G-250
CE: Cystic echinococcosis
EgAgB: *Echinococcus granulosus* antigen B
EgAg5: *Echinococcus granulosus* antigen 5
HRP: Horse-radish peroxidase
HF: Hydatid fluid
HLBP: Hydrophobic-ligand-binding-protein
IPG: Immobilized pH gradient
MALDI-TOF-MS: Matrix-assisted laser desorption/ioninzation time-of-flight mass spectrometry
nano-LC-ESI-MS/MS: Nano-liquid chromatography-electrospray ionization/multi-stage MS
ECL: SuperSignal chemiluminescence
PMF: Peptide mass fingerprint
2-DE: Two-dimensional electrophoresis
US: Ultrasonography
