# Supplementary material for: Alteration of immunoproteome profile of Echinococcus granulosus hydatid fluid with progression of cystic echinococcosis
Source: Parasit Vectors. 2015 Jan 8;8:10. doi: 10.1186/s13071-014-0610-7 (PMC4311513; doi:10.1186/s13071-014-0610-7)
Supplement: Additional file 1: — Demographic and clinical features of CE patients enrolled in this study. [file 13071_2014_610_MOESM1_ESM.docx]

**Additional file 1. Demographic and clinical features of CE patients enrolled in this study**

| CE stages  (no. used) | Age  (ranges) | Sex  (% male) | Cyst location  (no. of patients) | Representative ultrasound |
| --- | --- | --- | --- | --- |
| CE1 (11) | 29.6 ± 20.7  (12-88) | 45.5 | Right lobe (7)  Left lobe (3)  Multiple (1) | 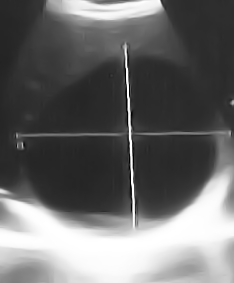 |
| CE2 (12) | 32.9 ± 16.4  (10-62) | 41.7 | Right lobe (7)  Left lobe (5) | 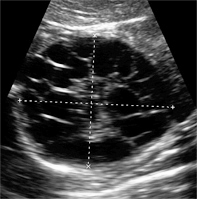 |
| CE3 (12) | 37.3 ± 24.5  (7-66) | 25 | Right lobe (5)  Left lobe (2)  Multiple (5) | 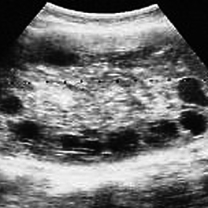 |
| CE4 (13) | 39.4 ± 18.9  (12-59) | 46.2 | Right lobe (8)  Left lobe (2)  Multiple (3) | 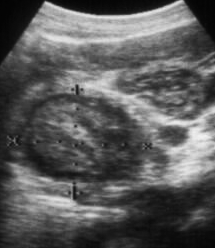 |
| CE5 (11) | 49.8 ± 14.7  (23-71) | 27.3 | Right lobe (7)  Left lobe (2)  Multiple (2) | 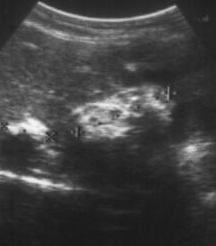 |
