# Supplementary material for: Alteration of immunoproteome profile of Echinococcus granulosus hydatid fluid with progression of cystic echinococcosis
Source: Parasit Vectors. 2015 Jan 8;8:10. doi: 10.1186/s13071-014-0610-7 (PMC4311513; doi:10.1186/s13071-014-0610-7)
Supplement: Additional file 2: — Protein identification of sheep CE1 and CE2 HF following MALDI-TOF-MS analysis. [file 13071_2014_610_MOESM2_ESM.docx]

**Additional file 2. Protein identification of sheep CE1 and CE2 HF following MALDI-TOF-MS analysis^a^**

| Spot  no. | MS^b^ | MP/  TP^c^ | SC^d^ | Theoretical Mw/pI | Accession no. | Description | SP^e^ |
| --- | --- | --- | --- | --- | --- | --- | --- |
| 1 | 259 | 26/36 | 45 | 79457/6.41 | XP_004003379 | Serotransferrin | + |
| **2** | **243** | **28/43** | **57** | **55590/8.39** | **CBH36490** | **PEPCK^f^** | **-** |
| 3 | 189 | 27/75 | 40 | 79457/6.41 | XP_004003379 | Serotransferrin | + |
| 4 | 93 | 15/75 | 31 | 53075/6.32 | XP_004002539 | Selenium-binding protein 1 | - |
| 5 | 48 | 4/18 | 20 | 51566/5.65 | CAA49451 | Immunoglobulin γ1 chain | - |
| 6 | 73 | 6/15 | 26 | 51419/5.65 | S31459 | Immunoglobulin γ1 chain, fragment | - |
| **7** | **154** | **14/32** | **46** | **38954/6.82** | **EgrG_000790200** | **Cathepsin B** | **+** |
| 8 | 78 | 13/56 | 36 | 60174/6.10 | XP_004018123 | Fumarylacetoacetase | - |
| **9** | **171** | **16/32** | **51** | **38954/6.82** | **EgrG_000790200** | **Cathepsin B** | **+** |
| **10** | **60** | **8/23** | **32** | **51378/8.26** | **EgrG_001028500** | **Citrate synthase** | **-** |
| **11** | **118** | **16/75** | **53** | **44227/8.03** | **EUB59398** | **Phosphoglycerate kinase** | **-** |
| 12 | 79 | 11/75 | 40 | 38645/7.14 | XP_004010700 | Sorbitol dehydrogenase | - |
| 13 | 115 | 15/75 | 55 | 38645/7.14 | XP_004010700 | Sorbitol dehydrogenase | - |
| 14 | 97 | 14/75 | 39 | 37895/6.75 | XP_004006414 | GPDH^g^ | **+** |
| 15 | 96 | 13/75 | 46 | 37895/6.75 | XP_004006414 | GPDH | **+** |
| 16 | 79 | 11/75 | 48 | 36852/6.80 | XP_004001969 | Alcohol dehydrogenase [NADP(+)] | - |
| 17 | 135 | 17/75 | 54 | 36852/6.80 | XP_004001969 | Alcohol dehydrogenase [NADP(+)] | - |
| 18 | 107 | 13/75 | 52 | 37106/7.12 | XP_004014363 | Dihydrodiol dehydrogenase 3-like | - |
| 19 | 72 | 10/75 | 38 | 37106/7.12 | XP_004014363 | Dihydrodiol dehydrogenase 3-like | - |
| 20 | 79 | 12/75 | 47 | 36994/8.12 | XP_004014369 | Dihydrodiol dehydrogenase 3-like | - |
| **21** | **72** | **10/75** | **45** | **33316/9.02** | **ADG65665** | **38 kDa EgAg5** | **-** |
| **22** | **121** | **15/75** | **42** | **39666/8.31** | **EgrG_000905600** | **Fructose 1,6 bisphosphate aldolase** | **-** |
| **23** | **122** | **9/23** | **33** | **33316/9.02** | **ADG65665** | **38 kDa EgAg5** | **-** |
| **24** | **88** | **7/20** | **27** | **33316/9.02** | **ADG65665** | **38 kDa EgAg5** | **-** |
| **25** | **131** | **12/38** | **47** | **33316/9.02** | **ADG65665** | **38 kDa EgAg5** | **-** |
| **26** | **136** | **13/52** | **48** | **33316/9.02** | **ADG65665** | **38 kDa EgAg5** | **-** |
| 27 | 98 | 12/75 | 47 | 34281/7.10 | XP_004012073 | Carbonic anhydrase 2 | + |
| 28 | 146 | 18/75 | 61 | 30693/8.55 | XP_004003446 | Carbonyl reductase [NADPH] 1-like | - |
| 29 | 135 | 16/75 | 59 | 30494/7.01 | XP_004003447 | Carbonyl reductase [NADPH] 1-like | - |
| 30 | 185 | 18/75 | 72 | 29726/7.70 | XP_004011906 | Carbonic anhydrase 3 | - |
| 31 | 78 | 13/42 | 17 | 93467/6.68 | XP_004020437 | γ-tubulin complex component 2 | - |
| **32** | **155** | **15/75** | **79** | **27123/6.60** | **EgrG_000416400** | **Triosephosphate isomerase** | **+** |
| 33 | 59 | 10/75 | 37 | 25869/7.66 | XP_004002338 | GST^h^ µ1, isoform 1 | - |
| 34 | 161 | 18/75 | 56 | 25385/8.87 | AAD42800 | microsomal GST | - |
| 35 | 134 | 17/75 | 54 | 25385/8.87 | AAD42800 | microsomal GST | - |
| 36 | 165 | 15/75 | 93 | 21117/6.96 | XP_004017427 | PEBP1^i^ | - |
| -37 | 77 | 10/73 | 74 | 23126/8.28 | XP_004019771 | GST-P | - |
| 38 | 197 | 18/75 | 90 | 21117/6.96 | XP_004017427 | PEBP1 | - |
| 39 | 171 | 16/75 | 89 | 21117/6.96 | XP_004017427 | PEBP1 | - |
| 40 | 164 | 15/34 | 66 | 22209/8.59 | XP_004001967 | Peroxiredoxin-1 isoform 1 | - |
| 41 | 96 | 7/19 | 48 | 22209/8.59 | XP_004001967 | Peroxiredoxin-1 isoform 1 | - |
| 42 | 79 | 5/11 | 43 | 15103/7.58 | NP_001138652 | FABP^j^, epidermal | - |
| **43** | **72** | **4/8** | **50** | **6232/8.31** | **ACZ51459** | **EgAgB1** | **+** |
| **44** | **88** | **4/8** | **50** | **6232/8.31** | **ACZ51459** | **EgAgB1** | **+** |
| **45** | **70** | **4/8** | **50** | **6232/8.31** | **ACZ51459** | **EgAgB1** | **+** |
| **46** | **67** | **4/8** | **50** | **6232/8.31** | **ACZ51459** | **EgAgB1** | **+** |
| **47** | **66** | **4/10** | **40** | **6232/8.31** | **ACZ51459** | **EgAgB1** | **+** |
| **48** | **69** | **4/9** | **44** | **6232/8.31** | **ACZ51459** | **EgAgB1** | **+** |
| **49** | **69** | **4/9** | **44** | **6232/8.31** | **ACZ51459** | **EgAgB1** | **+** |
| 50 | 98 | 9/25 | 30 | 26260/8.89 | XP_004010713 | Galectin-3 | - |
| 51 | 114 | 12/75 | 90 | 16050/6.49 | NP_001091117 | Hemoglobin subunit β | - |
| 52 | 100 | 11/75 | 84 | 16050/6.49 | NP_001091117 | Hemoglobin subunit β | - |
| 53 | 86 | 9/75 | 79 | 16050/6.49 | NP_001091117 | Hemoglobin subunit β | - |
| 54 | 90 | 10/75 | 83 | 14248/7.77 | XP_004005947 | FABP, liver | - |
| 55 | 80 | 9/75 | 53 | 15226/8.72 | CAA49751 | α-globin chain | - |
| 56 | 118 | 11/75 | 74 | 15226/8.72 | CAA49751 | α-globin chain | - |
| 57 | 133 | 12/75 | 78 | 15226/8.72 | CAA49751 | α-globin chain | - |
| **58** | **80** | **6/17** | **54** | **8337/6.78** | **ACZ51452** | **EgAgB4** | **+** |
| **59** | **96** | **40/78** | **54** | **8337/6.78** | **ACZ51452** | **EgAgB4** | **+** |
| **60** | **64** | **4/9** | **56** | **6232/8.31** | **ACZ51459** | **EgAgB1** | **+** |
| **61** | **73** | **4/17** | **50** | **6232/8.31** | **ACZ51459** | **EgAgB1** | **+** |
| **62** | **73** | **4/14** | **50** | **6232/8.31** | **ACZ51459** | **EgAgB1** | **+** |
| **63** | **48** | **3/13** | **39** | **6232/8.31** | **ACZ51459** | **EgAgB1** | **+** |
| **64** | **73** | **4/6** | **56** | **6232/8.31** | **ACZ51459** | **EgAgB1** | **+** |
| **65** | **81** | **5/10** | **67** | **6232/8.31** | **ACZ51459** | **EgAgB1** | **+** |
| **66** | **129** | **9/15** | **75** | **7223/9.23** | **ACZ51458** | **EgAgB1** | **+** |
| **67** | **98** | **7/25** | **67** | **6232/8.31** | **ACZ51459** | **EgAgB1** | **+** |
| **68** | **60** | **4/16** | **56** | **6232/8.31** | **ACZ51459** | **EgAgB1** | **+** |
| **69** | **46** | **3/8** | **39** | **6232/8.31** | **ACZ51459** | **EgAgB1** | **+** |
| **70** | **88** | **5/8** | **67** | **6232/8.31** | **ACZ51459** | **EgAgB1** | **+** |
| **71** | **62** | **4/11** | **56** | **6232/8.31** | **ACZ51459** | **EgAgB1** | **+** |
| **72** | **42** | **3/13** | **47** | **5348/9.30** | **ACP21247** | **EgAgB1 subunit, partial** | **+** |
| **73** | **114** | **8/18** | **75** | **7223/9.23** | **ACZ51458** | **EgAgB1** | **+** |
| **74** | **80** | **6/33** | **51** | **6232/8.31** | **ACZ51459** | **EgAgB1** | **+** |
| **75** | **80** | **6/33** | **51** | **9282/9.38** | **AAW78457** | **EgAgB2** | **+** |
| **76** | **96** | **6/11** | **44** | **9314/9.38** | **AAP83174** | **EgAgB subunit 2** | **+** |
| **77** | **96** | **4/14** | **50** | **6232/8.31** | **ACZ51459** | **EgAgB1** | **+** |
| **78** | **65** | **4/11** | **50** | **6232/8.31** | **ACZ51459** | **EgAgB1** | **+** |

^a^Parasite proteins are bolded. Host proteins were identified from sheep NCBI database.

^b^Mascot score

^c^Matched peptides/Total peptides.

^d^Sequence coverage (%).

^e^Signal peptide (SP) were predicted by the SignalP 4.1 and PSORT.

^f^Phosphoenolpyruvate carboxykinase

^g^Glycerol-3-phosphate dehydrogenase [NAD(+)], cytoplasmic isoform 2.

^h^Glutathione S-transferase

^i^Phosphatidylethanolamine-binding protein 1

^j^Fatty acid-binding protein
